# PReS13-SPK-1240: New aspects on APS pathogenesis

**DOI:** 10.1186/1546-0096-11-S2-I11

**Published:** 2013-12-05

**Authors:** PL Meroni

**Affiliations:** 1 Clinical Sciences and Community Health, University of Milan, Milan, Italy

## 

Antiphospholipid antibodies (aPL) are both diagnostic markers for, and pathogenic drivers of, antiphospholipid syndrome (APS). Beta 2 glycoprotein I (Beta2GPI)-dependent aPL, the most important subset, mediate different--and not necessarily alternative--thrombogenic mechanisms, mainly on the basis of their reactivity with Beta2GPI expressed on cells that participate in the coagulation cascade. *In vivo *experimental models showed that Beta2GPI cannot be detected in vascularized organs in resting conditions, while it can be over-expressed after pro-inflammatory stimulus. In this condition autoantibodies recognize the molecule, fix complement (C') and eventually induce clotting. This finding is in line with the observation that although the presence of aPL is a necessary pre-condition, APS-associated clotting is triggered by an additional 'second hit', frequently related to innate inflammatory immune responses.

Recurrent pregnancy complications associated with aPL cannot be explained solely by thrombosis, and alternative pathogenic mechanisms have been reported. Although one *in vivo *model of fetal loss supports a mechanism of aPL-mediated acute placental inflammation, other models and the histopathological examination of APS placentae do not support an inflammatory signature. Beta2GPI can be detected on endothelial cells of uterine vessels in resting conditions and its presence is increased during pregnancy in uterine endothelium and trophoblast. This finding does support the hypothesis that Beta2GPI -dependent aPL recognize their antigen on placental tissues. There is evidence that the antibodies may inhibit the growth and differentiation of trophoblasts, and eventually cause defective placentation so explaining the APS obstetric manifestations.

Why antibodies with similar antigen specificity produce different clinical manifestations is not clear. The formation of immune complexes on the membrane of cells with different biological functions may at least in part explain the diverse effects mediated by the autoantibodies (i.e. clotting versus defective placentation). Altogether this finding strongly supports the pivotal role of Beta2GPI as the main tissue target for aPL. Accordingly, new approaches have been tried in order to interfere with the antibody binding and its consequences. A synthetic peptide displaying a similarity in the PL-binding region of the V^th ^domain of the Beta2GPI was shown to compete with the molecule binding to the membrane of cells involved in the pathogenesis of the syndrome (i.e. endothelial cells, monocytes and trophoblasts). The passive infusion of the peptide was protective against the effect of polyclonal human APS IgG fractions on experimental thrombus formation and fetal loss induction. Moreover, a human monoclonal antibody against the immunodominant epitope of the Beta2GPI (Domain I) was shown to induce fetal loss and to trigger clotting by fixing C' in naïve mice. A similar monoclonal antibody lacking the CH2 fragment in the Fc gamma portion was still reacting with the Beta2GPI but no more able to activate C'. The C' non-fixing monoclonal antibody was protective by competing with the pathogenic one when passively infused in naïve mice and evaluated for both the induction of thrombus formation and the induction of fetal loss.

## Disclosure of interest

None declared.

